# Antimicrobial Activity and Molecular Mechanism of the CRES Protein

**DOI:** 10.1371/journal.pone.0048368

**Published:** 2012-11-21

**Authors:** Li Wang, Qing Yuan, Sunhong Chen, Heng Cai, Meige Lu, Yue Liu, Chen Xu

**Affiliations:** 1 Department of Histology and Embryology, Shanghai Jiaotong University School of Medicine, Shanghai, China; 2 Shanghai Key Laboratory of Reproductive Medicine, Shanghai, China; Clermont-Ferrand University, France

## Abstract

Cystatin-related epididymal spermatogenic (CRES) protein, a member of the cystatin superfamily of cysteine protease inhibitors (also known as CST8), exhibits highly specific, age-dependent expression in mouse testis and epididymis. The CRES protein possesses four highly conserved cysteine residues which govern the overall conformation of the cystatins through the formation of two disulfide bonds. Previous studies have revealed that other cystatin family members, such as cystatin 3 and cystatin 11, show antibacterial activity *in vitro*. This prompted us to investigate the potential antimicrobial activity of the CRES protein. Colony forming assays and spectrophotometry were used to investigate the effects of recombinant CRES protein on *Escherichia coli* (*E. coli*) and *Ureaplasma urealyticum* (*Uu*), respectively, *in vitro*. After incubation of *E. coli* with CRES recombinant protein fused with glutathione-S-transferase (GST), a substantial decrease in colony forming units was observed, and the effect was dose and time dependent. Furthermore, it took longer for *Uu* to grow to plateau stage when incubated with GST-CRES recombinant protein compared with the control GST. The antibacterial and Anti-*Uu* activities were not impaired when the cysteine residues of CRES protein were mutated, indicating that the antimicrobial effect was not dependent on its disulfide bonds. Functional analysis of three CRES polypeptides showed that the N-terminal 30 residues (N30) had no antimicrobial activity while N60 showed similar activity as full-length CRES protein. These results suggest that the active center of CRES protein resides between amino acid residues 31 and 60 of its N-terminus. Mechanistically, *E. coli* membrane permeabilization was increased in a dose-dependent manner, and macromolecular synthesis was inhibited on treatment with GST-CRES. Together, our data on the antimicrobial activities of CRES protein suggest that it is a novel and innate antimicrobial protein which protecting the male reproductive tract against invading pathogens.

## Introduction

Host defense proteins in plants and animals are vital components of the innate immune system and protect against the invasion of pathogenic microorganisms [1], [2], [3], [4], [5]. Recently, more and more studies have focused on host defense proteins in the male reproductive tract because of their potential roles in sexually transmitted diseases. The large number of peptides identified in recent years include the β-defensins (e.g. Defb41, Defb42 and Bin1b) [6], [7], [8], Cathelicidins (e.g. human cationic antimicrobial protein hCAP18) [9], [10], Lipocalins (e.g. prostaglandin D synthase), protease inhibitors (e.g. cystatin 3 and cystatin 11) [11], [12] and CXC chemokines (e.g. GCP-2/CXCL6, MIG/CXCL9) [13], [14], among others. These proteins together constitute a powerful protective barrier against bacteria, viruses, Mycoplasma, Chlamydia, and other pathogenic microorganisms; ensuring that sperm mature, and are transported and stored in a pathogen and disease-free environment. Among them, cystatin 3 (CST3) and cystatin 11 (CST11) belong to the CST type 2 family of cysteine protease inhibitors, which contains at least 10 members with widespread expression in all tissues and organs. However, 4 of them are specifically expressed in the male reproductive tract, including CST11, CRES (CST8), Testatin (CST9) and cystatin T (CST T), and are called the CRES subgroup [15]. Among them, CST11 is confined to the epididymis, especially at the initial segment. Importantly, CST11 possesses antimicrobial activities, which led us to hypothesize that CRES protein, a member of the same subgroup as CST11, may also have antimicrobial activity.

CRES gene was first identified from the mouse proximal caput epididymis by Cornwall in 1992, and according to sequence analysis, its encoded protein was a new member of the cystatin family. The amino acid sequence of the CRES gene is homologous to the cystatins in several regions of its sequence, as well as having four highly conserved cysteine residues in its C terminus which govern the overall conformation of the cystatins through the formation of two disulfide bonds [16]. However, unlike the widespread expression of the cystatins, CRES gene exhibited obvious tissue specific expression [16], [17], [18], [19]. Both Northern blot analysis and *in situ* hybridization demonstrated that CRES gene is expressed in testis, epididymis, ovary as well as pituitary gonadotropic hormone cells, but not in 26 other tissues including the deferent duct, adrenal gland and kidney. Also, the expression of CRES gene in testis and epididymis is much higher than that in ovary and the anterior pituitary, suggesting that the CRES gene may play a unique and tissue specific role in spermatogenesis and sperm maturation. CRES is expressed in a stage-specific manner in the testis. CRES mRNA was predominantly localized to the round and early elongating spermatids. CRES protein was first detected in the early elongating spermatids of Stages IX-XI, and reached the acrosome at the late stage. CRES protein localization became gradually limited to the anterior region of the sperm acrosome during their passage from the epididymal caput to the cauda [17], [20]. In the epididymis, CRES protein was only synthesized in the principal cells of the proximal caput epididymis and secreted into the lumen of the midcaput epididymis. CRES protein was absent from the distal caput epididymis, and undetectable in the epididymal corpus and cauda [17]. We have previously observed that the expression of CRES protein in testis and epididymis follows an apparent age-dependent pattern that is closely associated with key time points during spermatogenesis and sperm maturation [21]. The region-specific and age-dependent expression of the CRES protein is similar to that of another antibacterial peptide—Bin1b [22].

In the present study, we chose *Escherichia coli* (*E. coli*) and *Ureaplasma urealyticum* (*Uu*), two common pathogenic microorganisms in the male reproductive tract infection, to probe whether the CRES protein possesses antimicrobial activities. We determined whether the antimicrobial activity of CRES was dependent on its disulfide bonds by mutating the cysteine residues of the CRES protein. We then produced three N-terminal CRES polypeptides of different lengths, and measured their biological activities to determine the location of its active center. It has been reported that host defense proteins achieve their antimicrobial function via at least two distinct mechanisms. Several antimicrobial proteins and peptides exert their antibacterial effect by making use of their own cationic charge to attack bacterial membranes, which results in increased membrane permeability, an efflux of intracellular contents and eventual cell lysis [23]. The other antimicrobial mechanism involves the inhibition of the synthesis of macromolecular materials [24]. In this study, we investigated the antimicrobial mechanisms of the CRES protein by fluorescent staining and isotope labeling, in order to experimentally ascertain the biological functions of the CRES protein.

## Materials and Methods

### Ethics Statement

All experiments were conducted following the Guide for Care and Use of Laboratory Animals (the “NIH Guide”). The protocols for the use of animals were approved by the Department of laboratory animal sciences, Shanghai Jiao Tong University School of Medicine, and had the permit numbers SYXK (Hu) 2008 0050.

### Cloning and expression of mouse CRES fragments

**Figure 1:**
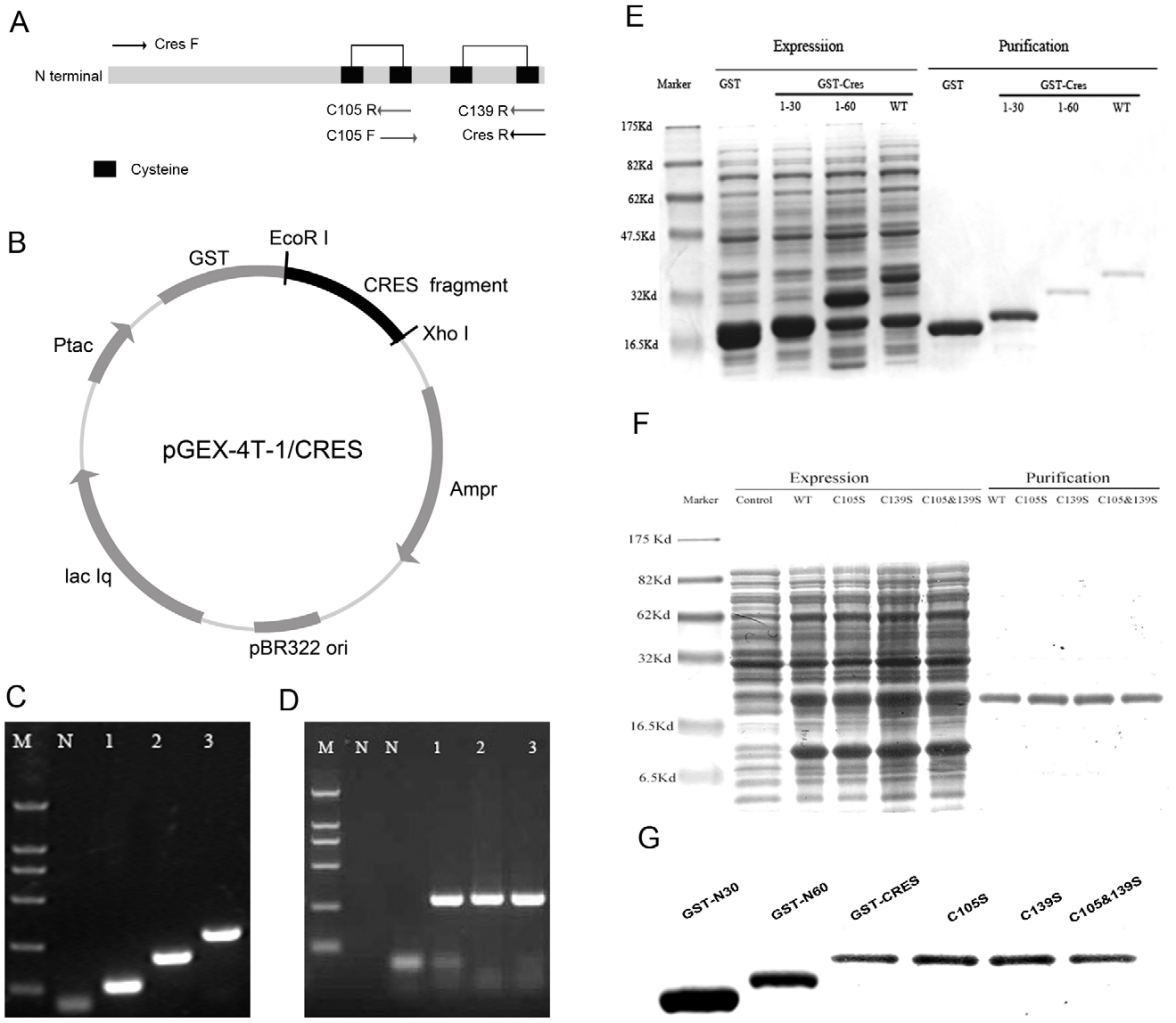
Cloning and expression of CRES. (A) Design of the oligonucleotide primers used to construct the cysteine mutants of CRES. (B) Schematic diagram of the recombinant plasmid PGEX-4T-1/CRES. (C) RT-PCR products of the CRES fragments on 1.5% agarose gel. Lane M, marker; lane N, negative control (PCR without cDNA); lane 1, CRES-N30 fragment; lane 2, CRES-N60 fragment; lane 3, CRES full length fragment. (D) RT-PCR products of the mutant CRES gene on 1.5% agarose gel. Lane M, marker; lane N, negative control; lane 1, CRES C105 mutant; lane 2, CRES C139 mutant; lane 3, CRES C105 & C139 mutant. (E) Prokaryotic expression and purification of CRES and GST recombinant protein. Lane M, marker; Lane 1, cells carrying the GST vector without the insert after Isopropylthio-β-D1-galctopyranoside (IPTG) induction; Lane 2–4, cells carrying the GST vector with the insert (CRES-N30,CRES-N60, full length CRES) after IPTG induction. Lane 5, purified GST recombinant protein. Lane 6–7, purified GST-CRES recombinant protein (N30, N60, N90). (F) Prokaryotic expression and purification of cysteine mutants of CRES. Lane M, marker; Lane 1, cells carrying the GST vector before IPTG induction; Lane 2–5, cells carrying the GST vector with the insert (wild type, C105 mutant, C139 mutant, C105 & C139 mutant) after IPTG induction. Lane 6–9, purified GST-CRES recombinant protein (wild type, C105 mutant, C139 mutant, C105 & C139 mutant). (G) The purified CRES recombinant proteins were analyzed by western blot using anti-GST monoclonal antibody.

Total RNA was extracted from C57BL/6 mouse epididymis using TRIzol (Invitrogen, U.S.A.), quantified with NanoDrop (Wilmington, DE, U.S.A.), and reverse-transcribed into first-strand cDNA using PrimeScript RT kit (TaKaRa, Da Lian, China). Then three segments of CRES were amplified by PCR using the following primers (Table 1). sense: CRES F; N30 segment antisense: N30R; N60 segment antisense: N60R; full length antisense: CRES R. The PCR products were cloned into pMD18-Tvector (TaKaRa, Kanazawa, Japan) and transformed in *E. coli* DH5α. The nucleotide sequences were confirmed by sequencing. These inserts were double digested and then inserted into the same double digested site of a GST-tag PGEX-4T-1 expression vector (Novagen Inc., Madison, Wisconsin, U.S.A.), (Fig. 1B). The open reading frame and identity of the insert in each construct were verified with plasmid sequencing. They were expected to produce progressively truncated proteins spanning: aa 1–30, aa 1–60, and aa 1–90. Recombinant expression plasmids, namely GST-N30, GST-N60, GST-CRES, were propagated in *E. coli* BL21 (DE3) host cells cultured with 0.1 mM isopropyl-β-D1-thiogalactopyranoside (IPTG) for 12 hours at 16°C with gentle shaking. Recombinant proteins were purified using a glutathione–sepharose resin affinity column (Pharmacia, U.S.A.). A GST vector lacking the cDNA insert was expressed and purified as a control. The molecular weight (kDa) of the expressed proteins was verified by Coomassie blue staining after sodium dodecyl sulfate–polyacrylamide gel electrophoresis and Western blots using anti-GST monoclonal antibody (Kangwei, Beijing, China).

**Table 1:** The primer sequences used to amplify the CRES protein fragments and to construct the cysteine mutants of CRES.

1	CRES F	GGAATTCatgaaagaatacaacaaggaaagtg
2	N30 R	CCGCTCGAGttccattcggtctgtgatctgaag
3	N60 R	CCGCTCGAGcagttcgggttttttttgagggatg
4	CRES R	CCGCTCGAGttagacatctttacattctttgctc
5	C105 F	caatactgaaaactccatccctc
6	C105 R	gagggatggagttttcagtattg
7	C139 R	CCGCTCGAGttagacatctttagattctttgctc

The base in red is the mutational site. CTCGAG: XhoI site, GAATTC: EcoRI site.

### Cloning and expression of cysteine mutants of CRES

The single cysteine mutants (Cys-105→Ser or Cys-139→Ser) and double cysteine mutant (Cys-105→Ser and Cys-139→Ser) of CRES were performed utilizing site-directed mutagenesis [25], [26] using two separate PCR reactions with a single mutagenic primer and two vector-specific primers. The oligonucleotides used for constructing the mutants were designed according to Fig. 1A, and the concrete sequences were listed in Table 1. After sequencing the DNA regions containing the codon changes, the mutant gene fragments were transferred into the expression vector PGEX-4T-1 (Fig. 1B). Expression and purification of the site-specific mutants of CRES were conducted as described above.

### Antibacterial assay

The antibacterial activity of recombinant CRES protein and its cysteine mutants were determined by the CFU assay as described previously [12], [27]. Briefly, *E. coli* DH5α were grown to mid-log phase (A600, 0.4∼0.6). Approximately 10^3^ CFU bacteria were incubated at 37°C with recombinant CRES or its mutant proteins, and aliquots of the assay mixture were taken at 0.5, 1, 2 and 4 hours after incubation. Then the assay mixtures were serially diluted with 10 mM sodium phosphate buffer (pH 7.4) and 100 µl of each was plated on a Luria–Bertani agar plate and incubated at 37°C overnight. Recombinant GST protein was used as a negative control. Antibacterial activity expressed as percentage survival was calculated using the following formula: % survival = (number of colonies surviving after treatment with the antibacterial peptide/number of colonies surviving without the antibacterial peptide) ×100.

### Anti-*Uu* assay

Mouse recombinant CRES and its mutant proteins were tested for anti*-Uu* activity using a spectrophotometric method. Briefly, *Uu* were grown to mid-log phase and then 10 µl *Uu* were incubated at 37°C with recombinant CRES or its mutant proteins. The absorbance was measured at different times of incubation (0, 12, 18, 24 and 36 hours) at 550 nm. Recombinant GST protein was used as a negative control and the buffer used to dilute the CRES protein as a blank control.

### Epididymal epithelial cell (EEC) culture and cytotoxicity studies

The procedures for primary culture of mouse EECs were performed as previously described [28]. Briefly, disaggregated cells were obtained after successive enzymatic digestions. After differential adhesion for 4 hours, the epithelial cells were eventually collected and incubated at 37°C in 100% humidity and 5% CO_2_. Passage 3 cells were used for cytotoxicity studies. The EECs were seeded in 96 well plates and treated with varying concentrations (1 ng/mL, 10 ng/mL, 100 ng/mL, 1 µg/mL, 10 µg/mL) of recombinant CRES protein for 2 hours. The MTT assay was adopted to determine cytotoxicity of the CRES protein. The absorbance was monitored at 540 nm by Thermo Scientific Multiskan GO Microplate Spectrophotometer (Thermo Fisher, America). Recombinant GST protein was used as a negative control.

### 1-N-Phenyl-Naphthylamine (NPN) Uptake Assay

The ability of CRES recombinant protein to permeabilize the membrane of *E. coli* was examined using the 1-N-Phenyl-Naphtylamine (NPN) Uptake Assay Kit (GEMED SCIENTIFICS INC., Arlington, U.S.A.), as per manufacturer's instructions. Fluorescence was detected by a luminescence spectrometer (Perkin Elmer, U.S.A.) with excitation wavelength at 355 nm and emission wavelength at 460 nm.

### Transmission electron microscopy

Mid-log phase DH5α suspended in PBS were treated with 1 µg/mL CRES recombinant protein. After incubation of 2 hours, the bacterial cells were washed with PBS twice and then fixed with 4% glutaraldehyde overnight at 4°C. The follow-up sample preparation process was completed by the electron microscope laboratory of Shanghai Jiaotong University School of Medicine. Finally, the ultra thin sections were examined and photographed using CM120 transmission electron microscope (Philips, Holland).

### Macromolecular synthesis assay

Bacterial DNA synthesis affected by the CRES recombinant protein was tested by isotopic tracer method as described previously [24], [29]. Briefly, 10^6^ mid-log phase DH5α suspended in 1 ml PBS were treated with various concentrations of CRES recombinant protein (0, 0.1 µg/mL, 0.2 µg/mL, 0.5 µg/mL, 1 µg/mL, 1.5 µg/mL) and 2.5 µl [methyl-^3^H] thymidine (30 Ci/mmol), [5-^3^H]uridine (25.5 Ci/mmol) or L-[4, 5-^3^H (N)]leucine (20 Ci/mmol). After 2 hours, 10% ice-cold trichloroacetic acid was added to the bacterial suspensions, which were then placed on ice for 40 min. The bacterial suspensions were collected to glass microfiber filters by vacuum filtration and then washed twice each with 5% trichloroacetic acid and 70% ethanol. The microfiber filters were dried in an oven and transferred to 1 ml xylene containing 0.6% β-PBD. Finally the counts were obtained by LS6500 liquid scintillation counter (Beckman, U.S.A.) for 2 min for each filter.

### Statistical analysis

All assays were repeated at least three times. Results from the experimental and the control groups were recorded and analyzed using the SAS 8.2 statistical software. The results were analyzed by the Student's *t* test and differences were reported at *p*<0.05 as the level of significance.

## Results

### Cloning and expression of the CRES protein fragments and cysteine mutants

The CRES protein fragments and cysteine mutants were amplified from *Mus musculus* cDNA by RT-PCR and separated on a 1.5% agarose gel (Fig. 1C–D). The recombinant protein fragments were successfully expressed as the recombinant fragments fused with GST-tag in *E. coli* BL21 (DE3) and purified under denaturing conditions and then confirmed by 15% SDS-PAGE analysis and Western blots using an anti-GST monoclonal antibody (Fig. 1E–G). The molecular weights (MW) of CRES proteins without the GST-tag as predicted by DNASTAR are as follows: N-30: 3.686 KD, N-60: 7.186 KD, CRES: 10.555 KD, CRES-C105S: 10.555 KD, CRES-C139S: 10.555 KD, CRES-C105&139S: 10.555 KD. The MW of the GST-tag is 26 KD. We confirmed that the fusion proteins were in-frame with the GST-tag and the actual MWs were consistent with what was predicted. (GST-N30, 30 KD; GST-N60, 33 KD; GST-CRES, 37 KD; GST-C105S, 37 KD; GST-C139S, 37 KD; GST-C105/139S, 37 KD).

### Antibacterial activity

**Figure 2:**
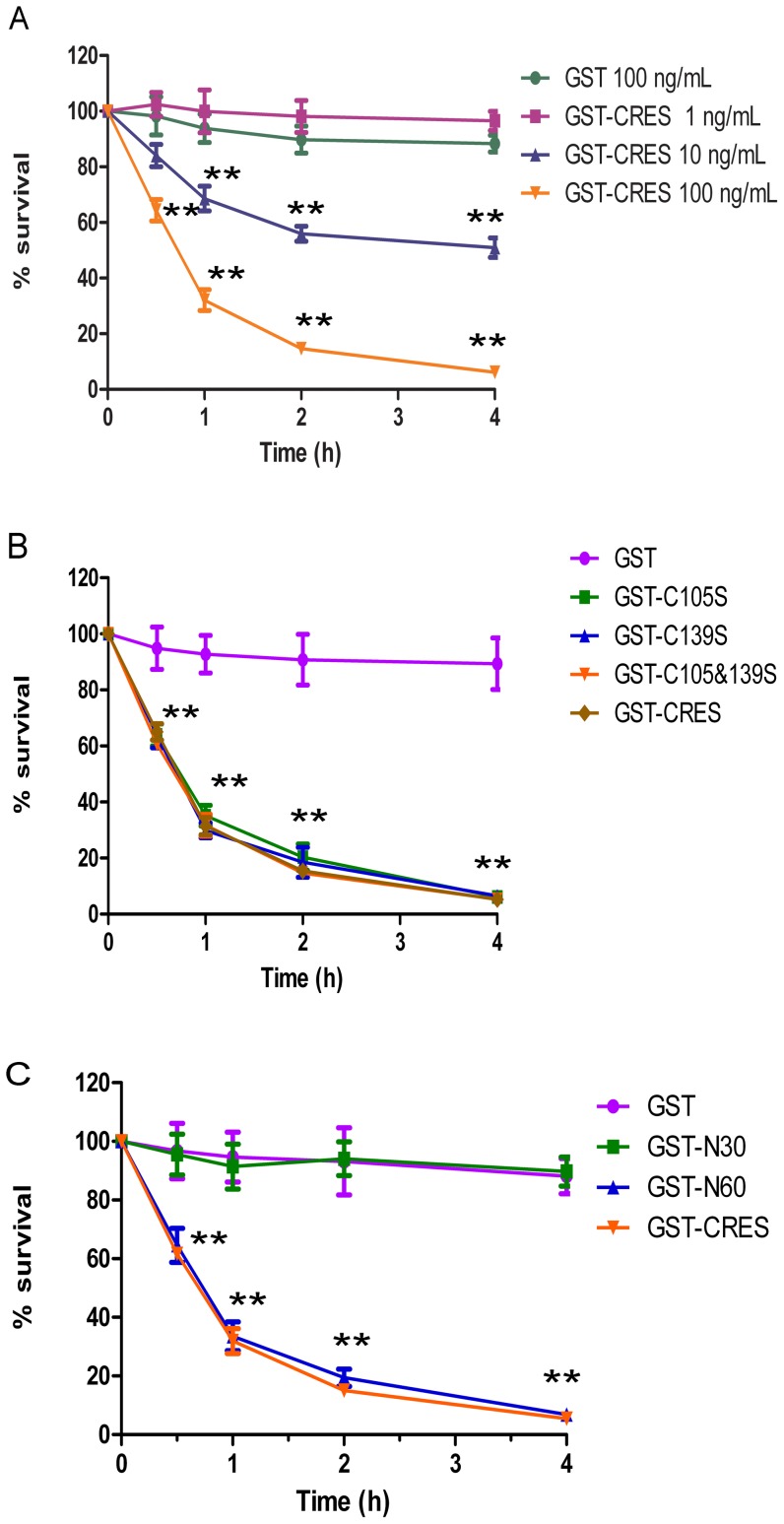
Antibacterial activities of CRES. (A) *E. coli* D5α were incubated with 1 ng/mL (purple), 10 ng/mL (blue), 100 ng/mL (orange) GST-CRES and 100 ng/mL GST (green) for 0.5 h, 1 h, 2 h, and 4 h. (B) *E. coli* D5α incubated with 100 ng/mL GST (purple), C105 mutant (green), C139 mutant (blue), C105 & C139 mutant (orange), GST-CRES (brown) for 0.5 h, 1 h, 2 h, and 4 h. (C) *E. coli* D5α incubated with 100 ng/mL GST (purple), GST-N30 (green), GST-N60 (blue), GST-CRES (orange) for 0.5 h, 1 h, 2 h, and 4 h. ** *p*<0.01.

Antibacterial assays were performed to assess the activity of the CRES protein fragments and cysteine mutants against *E. coli* DH5α. As shown in Figure 2A, the full length GST-CRES protein exhibited antibacterial activity in a dose and time- dependent manner. The survival of *E. coli* DH5α was decreased at CRES concentrations of 100 ng/mL and 10 ng/mL (*p*<0.01), but no apparent change was found at the lower concentration of 1 ng/mL. The GST recombinant protein showed no detectable antibacterial activity when incubated for 4 hours at a concentration of 100 ng/mL.

The single cysteine mutants (Cys-105→Ser or Cys-139→Ser) and double cysteine mutant (Cys-105→Ser and Cys-139→Ser) of CRES exhibited obvious antibacterial activity similar to the full length CRES protein at the concentration of 100 ng/mL (Fig. 2B). An approximately 95% reduction in CFU was observed after incubation for 4 hours with 100 ng/mL full length CRES protein or mutant CRES proteins.

The CRES protein fragment N30 did not inhibit the growth of *E. coli* DH5α. However, the fragment N60 exerted the same strong antibacterial effect as the full length CRES protein (Fig. 2C).

### Anti-*UU* assay

**Figure 3:**
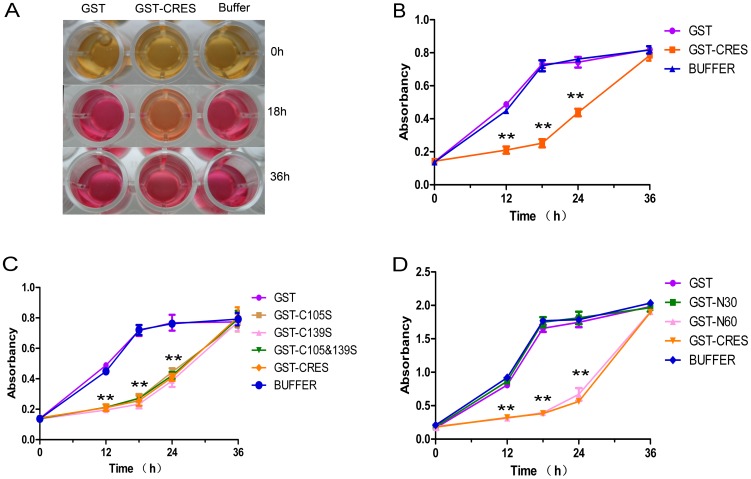
Anti-*Ureaplasma urealyticum* activities of CRES. (A) *Ureaplasma urealyticum (Uu*) incubated with 100 ng/ml GST, GST-CRES and buffer for 0 h, 18 h and 36 h. (B) Uu incubated with 100 ng/ml GST (purple), GST-CRES (orange) and buffer (green) for 0 h, 12 h, 18 h, 24 h and 36 h. (C) Uu incubated with 100 ng/ml GST (purple), C105 mutant (brown), C139 mutant (pink), C105 & C139 mutant (green), GST-CRES (orange) and buffer (blue) for 0 h, 12 h, 18 h, 24 h and 36 h. (D) Uu incubated with 100 ng/mL GST (purple), GST-N30 (green), GST-N60 (pink), GST-CRES (orange) and buffer (blue) for 0 h, 12 h, 18 h, 24 h and 36 h. ** *p*<0.01.

As shown in Figure 3A, the growth of *Uu* incubated with 100 ng/mL recombinant CRES protein reached a plateau after 36 hours, while it reached the plateau after 18 hours in the control group. There was a significant difference in the absorbance at 550 nm between samples incubated with GST-CRES and those incubated with buffer at 12, 18 and 24 hours (*p*<0.01), indicating that recombinant GST-CRES protein possesses anti-mycoplasma activity (Fig. 3B).

Similar to the full length CRES protein, the cysteine mutants of CRES could greatly inhibit the growth of *Uu*. The absorbance at 550 nm after incubation with mutants and buffer in 12, 18 and 24 hours were significantly different (*p*<0.01) (Fig. 3C).

The CRES protein fragment N60 presented powerful anti-*Uu* activity, just like the full length protein and as with *E. coli*. The N30 fragment exhibited no significant effects (Fig. 3D).

### MTT assay

**Figure 4:**
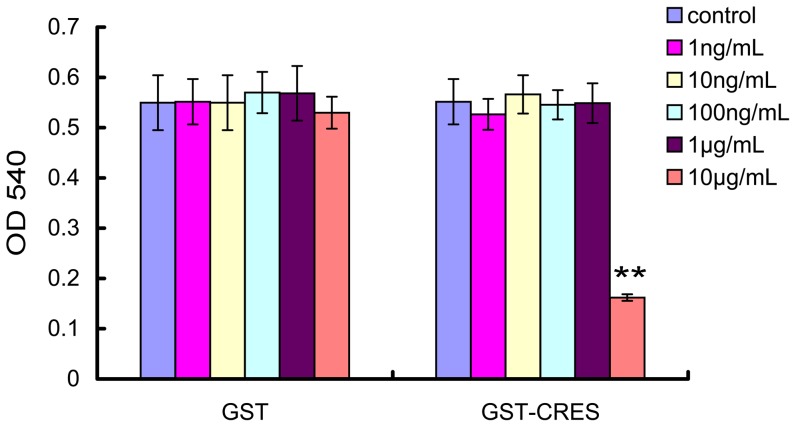
Determination of the epididymal epithelial cells viability treated with CRES using MTT assay. ** *p*<0.01.

As shown in Figure 4, CRES did not affect the viability of the epididymal epithelial cells (EECs) at concentrations from 1 ng/mL to 1 µg/mL. There were no significant differences between CRES treated groups and the control group at concentration from 1 ng/mL to 1 µg/mL (*p*>0.05). However, 10 µg/mL CRES inhibited the viability of EECs significantly (*p*<0.01).

### 1-N-Phenyl-Naphthylamine (NPN) Uptake Assay

**Figure 5:**
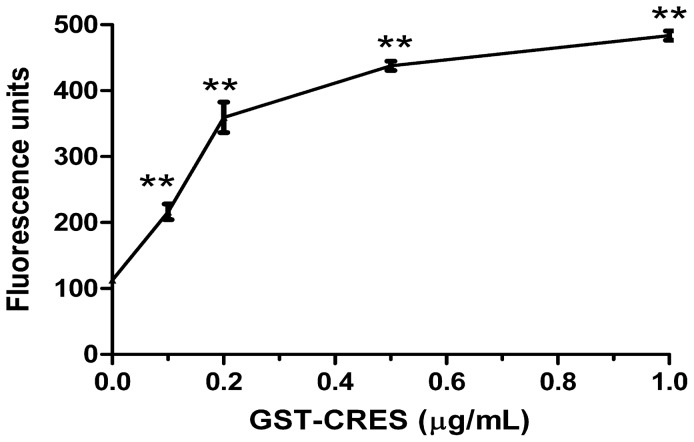
Permeabilization of bacterial membranes mediated by CRES. * *p*<0.05, ** *p*<0.01.

To investigate whether CRES recombinant protein affects *E. coli* membrane permeability, we used the NPN dye, which fluoresces strongly in phospholipid environments but only weakly in aqueous environments. Once the bacterial membrane is disrupted, the NPN dye enters the damaged membrane where it is strongly fluorescent. After treatment with CRES, a dose-dependent increase of fluorescence intensity was observed, which indicates that bacterial membrane permeabilization was increased (Fig. 5).

### Transmission electron microscopy

**Figure 6:**
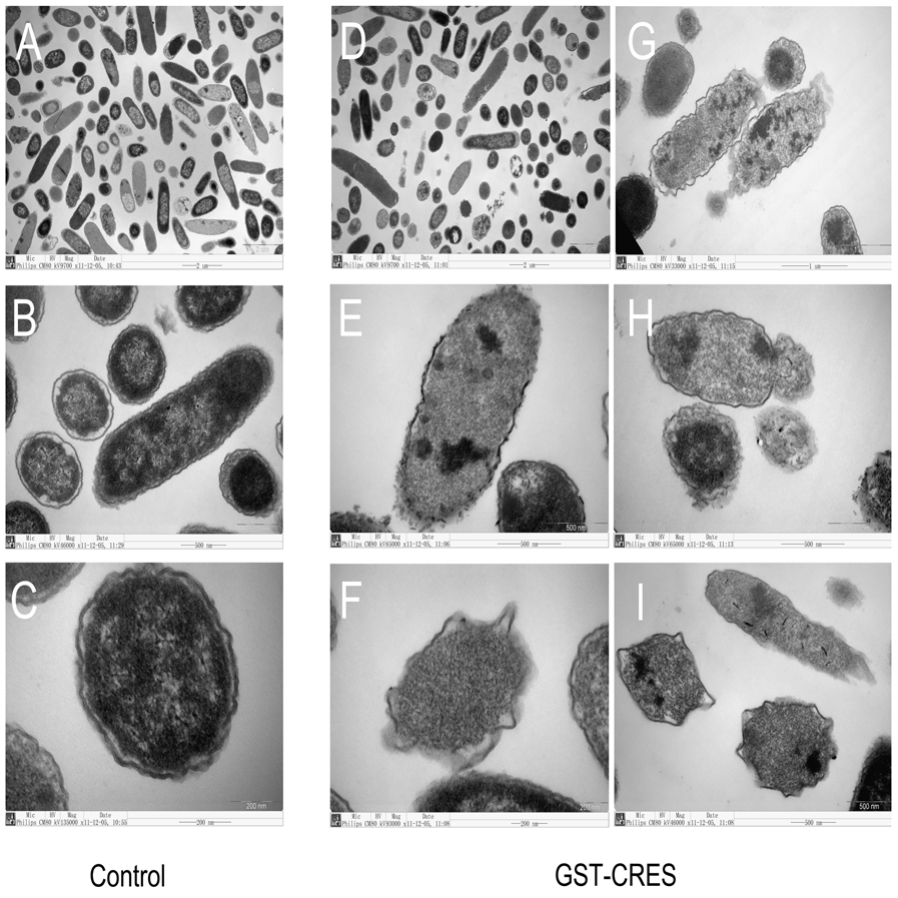
Transmission electron micrographs of *E. coli* D5α. (A–C) *E. coli* D5α incubated with buffer. (D–I) *E. coli* D5α incubated with GST-CRES.

Transmission electron microscopy was used to examine whether the CRES protein affected bacterial structure. As shown in Figure 6, the untreated *E. coli* DH5α exhibited an intact, smooth and continuous membrane structure (Fig. 6A-C). The membranes of *E. coli* DH5α treated with CRES had lost integrity, were discontinuous, and were clearly damaged. Furthermore, we found that bacterial contents had exuded from the damaged membrane (Fig. 6D–I).

### Macromolecular synthesis assay

**Figure 7:**
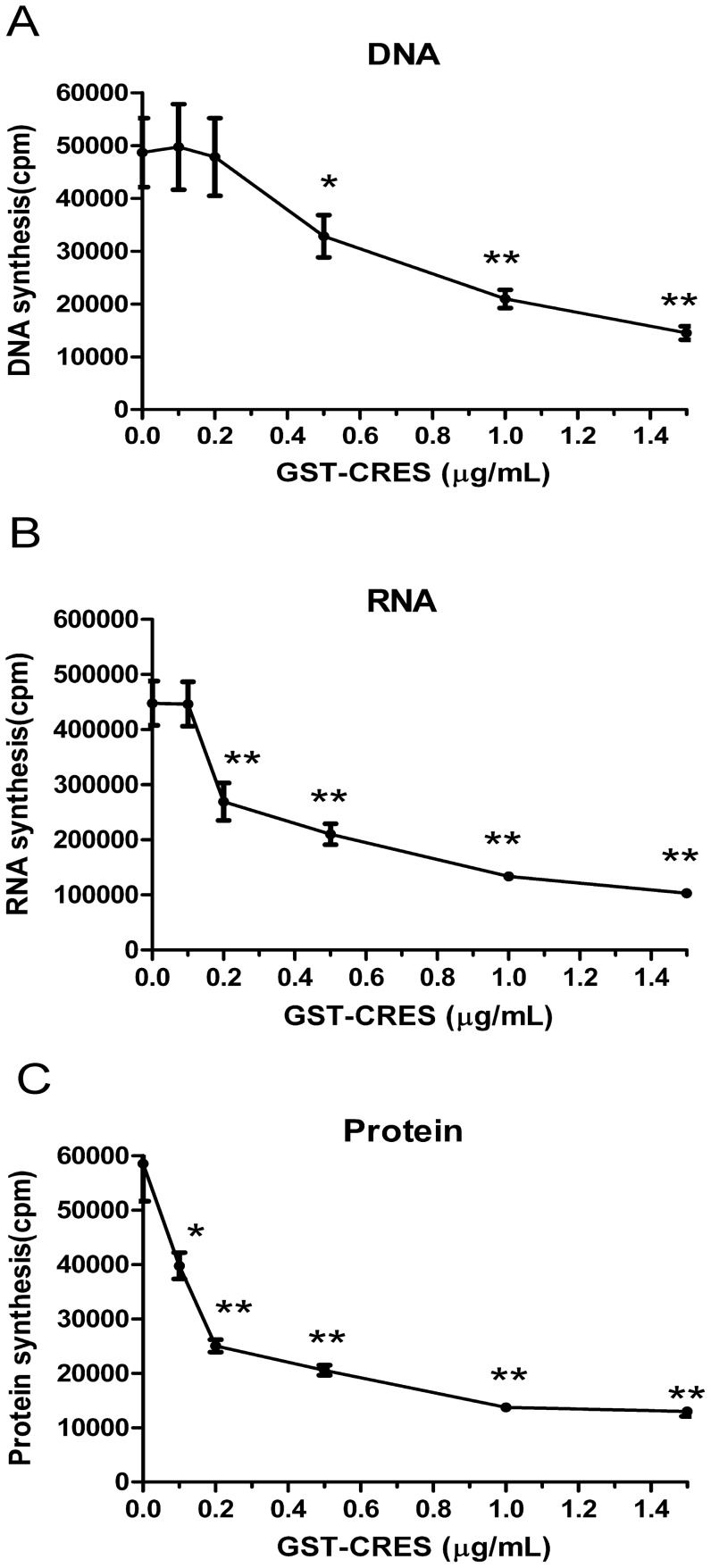
Macromolecular synthesis of *E. coli* D5α affected by CRES. (A) DNA synthesis. (B) RNA synthesis. (C) Protein synthesis. * *p*<0.05, ** *p*<0.01.

To determine the effects of CRES recombinant protein on macromolecular synthesis in *E. coli*, the incorporation of radioactive precursors [methyl-^3^H] thymidine, [5-^3^H] uridine and L-[4, 5-^3^H (N)] leucine into *E. coli* (DH5α) DNA, RNA and protein was examined after incubation for 2 hours in the presence of varying concentrations of CRES recombinant protein. We found that CRES inhibited DNA, RNA and protein synthesis in a dose-dependent manner (Fig. 7).

## Discussion

The infection and inflammation of the reproductive tract is a major concern for male reproductive health and contributes significantly to impaired fertility [29]. *E. coli* is one of the most common causative agents of epididymo-orchitis and is responsible for 65%–80% of total acute or chronic prostatitis cases [30], [31]. *Uu* is an opportunistic pathogenic agent, which can cause non-gonococcal urethritis (NGU) [32], prostatitis [33], and chorioamnionitis [34], under certain conditions. *E. coli* or *Uu* infection is closely associated with male infertility [30], [35], [36], [37]. In addition, bacterial strains resistant to conventional antibiotics have emerged in recent decades, resulting in significant additional financial burden, and in increase in mortality rates. Therefore, there is an urgent need to develop novel antimicrobial therapies to combat these pathogens. Antimicrobial peptides have long been suggested as a promising and novel therapeutic approach for drug-resistant infections [2], [38], [39]. In this study, we demonstrated that the CRES protein possessed anti-*E. coli* activity and that the effects were time and dose dependent. Furthermore, we found that the CRES protein could also inhibit the growth of *Uu*. However, it still needs some other researches to determine whether the concentrations of CRES shown to antimicrobial are physiologically relevant, because it has not been reported about the physiological concentration of CRES. CRES did not affect EEC viability at concentrations from 1 ng/mL to 1 µg/mL. But 100 ng/mL of CRES inhibited the growth of *E. coli* and *Uu*. Therefore, 100 ng/mL of CRES may be an optimal dose based on its potent antimicrobial effects, as well as the negligible toxicity associated with it.

Most antimicrobial peptides are very small and strongly cationic [2]. Some structural characteristics of the CRES protein that suggest that it is an antimicrobial peptide include: 1) small size, the protein encoded by small transcript only concluded 90 amino acid 2) contains two disulfide bonds; 3) theoretical pI: 8.55 (calculated according to its amino acid sequence by DNASTAR software)>pH, which suggests that CRES may be a cationic peptide. Therefore, CRES is potentially a new and innate cationic antimicrobial peptide produced in the male reproductive tract. Since the broad-spectrum of antimicrobial effects of CRES remain undetermined, our results present a novel target for research on the prevention and treatment of male reproductive tract infections. Our results have implications for reducing male infertility, and promoting male reproductive health.

The CRES protein contains 4 cysteine residues, which are assumed to form two disulfide bonds that maintain its three dimensional structure. In previous studies, the disulfide bonds were found to be essential for the function of antimicrobial peptides. The antimicrobial activities of several antimicrobial proteins were reduced or abolished by destruction of their disulfide bonds, such as dodecapeptide [40] and human epididymis 2 (HE2) [27]. However, we found that cysteine mutants of CRES protein displayed similar antimicrobial function to the wild-type CRES protein, suggesting that antimicrobial activities of CRES were not dependent on its disulfide bonds. We also found that CRES fragment N60 exhibited antimicrobial activities similar to the full length CRES protein. Furthermore, the second disulfide bond was located between N60 and N90. These data indicate that disulfide bonds are not necessary for CRES antimicrobial function. The antimicrobial activity of the CRES fragment N30 was not significantly different from that of the control peptide, while N60 showed similar activity as full-length CRES protein. These results indicate that the active center of CRES protein resides between amino acid residue 31 and 60 in its N-terminus. The precise location of the active center of CRES protein, however, requires further characterization.

Finally, we explored the antibacterial mechanism of CRES protein. CRES killed *E. coli* by permeabilizing and disrupting *E. coli* membranes, and inhibited macromolecular synthesis to block bacterial growth, consistent with previous findings [27], [29]. Cornwall et al demonstrated the CRES protein can self-aggregate and form amyloid structures *in vitro* and *in vivo* [41], [42]. They reported that the amyloid structures containing CRES were a component of the normal mouse epididymal lumen and exerted no apparent cytotoxic effects on spermatozoa [42]. Since amyloids are cytotoxic by virtue of their ability to form pores or channels in cell membranes [42], we speculate that the presence of functional amyloids containing CRES in the epididymal lumen may be associated with the antimicrobial effects of CRES.

Taken together, we have demonstrated for the first time that the mouse CRES protein is an innate antimicrobial peptide of the male reproductive tract, which may play an essential role in protecting the male reproductive tract against the invasion of pathogenic microorganisms. Our results may help to elucidate the biological function of the CRES protein and provide new approaches for the prevention and therapy of male reproductive tract infection.
